# Cellular shortening and calcium dynamics are improved by noisy stimulus in a model of cardiomyopathy

**DOI:** 10.1038/s41598-023-41611-6

**Published:** 2023-09-09

**Authors:** Russell Morales-Rubio, Judith Bernal-Ramírez, Nestor Rubio-Infante, Luis A. Luévano-Martínez, Amelia Ríos, Bruno A. Escalante, Gerardo García-Rivas, Jesús Rodríguez González

**Affiliations:** 1 Centro de Investigación y de Estudios Avanzados del I.P.N-Unidad Monterrey, Vía del Conocimiento 201, Parque de Investigación e Innovación Tecnológica, 66600 Apodaca, NL México; 2 Tecnologico de Monterrey, Escuela de Medicina y Ciencias de la Salud, Cátedra de Cardiología, Hospital Zambrano Hellion, TecSalud, San Pedro Garza García, México; 3 The Institute for Obesity Research, Tecnologico de Monterrey, Monterrey, Mexico

**Keywords:** Systems biology, Cellular noise, Dynamical systems, Heart failure

## Abstract

Noise is present in cell biology. The capability of cells to respond to noisy environment have become essential. This study aimed to investigate whether noise can enhance the contractile response and Ca^2+^ handling in cardiomyocytes from a cardiomyopathy model. Experiments were conducted in an experimental setup with Gaussian white noise, frequency, and amplitude control to stimulate myocytes. Cell shortening, maximal shortening velocity, time to peak shortening, and time to half relaxation variables were recorded to cell shortening. Ca^2+^ transient amplitude and raise rate variables were registered to measure Ca^2+^ transients. Our results for cell shortening, Ca^2+^ transient amplitude, and raise rate suggest that cell response improve when myocytes are noise stimulated. Also, cell shortening, maximal shortening velocity, Ca^2+^ transient amplitude, and raise improves in control cells. Altogether, these findings suggest novel characteristics in how cells improve their response in a noisy environment.

## Introduction

Excitation–contraction coupling (ECC) is a physiological process that transforms action potential into contractile force. The process involves a series of chemical reactions that converts the calcium ions inside the cardiomyocyte into mechanical movement^1,2^. Intracellular Ca^2+^ is an important secondary messenger for ECC. Anticancer therapies could induce alterations in Ca^2+^ handling^3^.

Doxorubicin (DOX) is an anthracycline drug used as a chemotherapeutic to treat numerous cancers, including leukemia, lymphomas, and solid tumors^4^. Despite the highly effective antineoplastic DOX properties, one side effect that limits its clinical utility is the dose-dependent cardiotoxicity caused by the drug, which can lead to irreversible cardiomyopathy and subsequent heart failure^5^. Thus, the doxorubicin-induced cardiomyopathy (DIC) murine model is characterized by systolic dysfunction and Ca^2+^ dysregulation, such as a decrease in the intracellular Ca^2+^ transient amplitude and decay rate and increased diastolic Ca^2+^ levels; as well as reduced sarcoplasmic reticulum Ca^2+^ ATPase (SERCA), and ryanodine receptors (RYR2) expression^6^. In addition, multiple mechanisms are associated with DIC, including increased reactive oxygen species production (ROS), disruption of Ca^2+^, and mitochondrial bioenergetics^7^.

One of the major challenges in modern biology is to understand how a cell operates in a highly noisy environment^8^. Physiological systems are permeated by noise and variability at all observational levels, ranging from molecular processes to complex interactions and dynamics of tissues, organs, and organisms^9^. Noise is often regarded as an unwanted system component, even though it sometimes enhances system performance in medicine and biology^10^. Stochastic resonance describes the positive impact of noise in nonlinear systems^11^. For instance, stochastic noise might provide new treatment alternatives to enhance vestibular function in patients with vestibular pathology^12^. External noise enhances sensory perception in primary neurons^13^. In the cardiovascular system at the whole organ scale, synchronization between heart contraction and external electrical stimuli is improved by noise^14^. A positive effect of noise in promoting skeletal muscle regeneration in neuropathological disease condition was studied three years ago^15^. Low-intensity noise favors calcium dynamics in H9c2 cells^16^.

In summary, there has been substantial progress in understanding the effects of noise on physiological systems. However, understanding how cells of a heart with cardiomyopathy respond to noisy stimuli has received little attention. The present work is devoted to partially filling this gap in knowledge. We address a key question: How does noise in the cellular environment affect the response of cardiomyocytes with impaired contractile response and Ca^2+^ handling? Our main goal is to understand whether cell shortening, and Ca^2+^ dynamics responses are improved when myocytes are noise stimulated. To this end, we analyzed shortening and Ca^2+^ dynamics in cardiomyocytes in a short-time mouse model of DOX-induced cardiomyopathy.

## Methods

All animal experimental procedures were conformed to the National Institutes of Health Guide for the care and use of laboratory animals (1996) and were approved by institutional Ethics Review Committee for Animal Experimentation of Cinvestav-IPN (approval 0170-15). Male C57B1/6j mice (30–40 g weight) were obtained from the experimental Animal Care center from CINVESTAV-IPN Mexico. Mice were acclimated for one week before experiments in metabolic cages (Nalgene, USA), and maintained in a temperature-controlled room (22° ± 2 °C) on a 12 h light–dark cycle (50%) fed a balanced diet and had water ad libitum. Experimental protocols were designed according to the Directrices from *the National Centre for the replacement Refinement & Reduction of Animal Research*, ARRIVE guidelines. A total of 50 mice were divide in four groups: DOX produces myocyte atrophy (DOX n = 6, CTRL n = 4), DOX effect in cell shortening and calcium fluxes (DOX n = 8, CTRL n = 6), mitochondrial function (DOX n = 7, CTRL n = 5), effect of noisy stimulus on cellular shortening and calcium fluxes (DOX n = 8, CTRL n = 6). In general, 12 mice were studied in each group, except in some non-invasive experiments where projections of sample size from available studies allowed us to use a smaller number of animals. Non-responding mice were dismissed.

### DOX treatment

Cardiac dysfunction model was established based on previous literature^17^. Mice received a total of 25 mg/kg of DOX (Sigma-Aldrich, St. Louis, Mo, USA) suspended in saline solution, administrated periodically in five intra-peritoneal injections of 5 mg/kg weekly/once a week. The control group received saline solution injections (NaCl solution 0.9%). Experiments were performed one week after the last intra-peritoneal injection.

### Cardiac function by Doppler measurements

Mice were anesthetized by mask inhalation of isoflurane vaporized at concentrations of up to 3%, while taped to a temperature-controlled laminated plastic board. Electrodes were placed in such a way that all four limb leads could be used for electrocardiographic monitoring. Aortic and mitral blood flow velocities were measured from the apical view using a 2-mm diameter 10-MHz pulsed Doppler probe and a real-time Doppler spectrum analyzer probe (Indus Instruments, Houston, TX, USA). The probe was placed just below the sternum using minimal pressure angled toward the ventricular inflow and outflow tracks. In real-time, the spectral Doppler signal was displayed along with the ECG on a computer monitor^18^. Signals were digitized and stored on a computer and analyzed offline.

### Histological assessments

Hearts were excised and weighted from each experimental group and fixed in 4% (wt/vol) paraformaldehyde in PBS at room temperature for 12 h. Afterward, tissues were embedded in paraffin and stained with hematoxylin/eosin (H&E) and Masson’s trichrome. Images were obtained in an Imager.M2 Zeiss microscope with an AxioCam HRm and analyzed with the AxioVision software. The micrographs from the whole of Masson’s trichrome slides were taken at 2.5×; then, images were decomposed in more than seven fields at 5×. The fibrotic index was assessed by quantification of blue and red pixels, using ImageJ (http://imagej.nih.gov/ij/, NIH, Bethesda, MD, USA); a % blue/% red ratio was made. Two blinded researchers analyzed the complete slides. H&E micrographs from the papillary muscles were used to quantify the cardiomyocyte area at 10×.

### BNP gene quantification

Total RNA from the tissue of the ventricles was isolated using a TRIzol Reagent (15596026, Invitrogen) to evaluate brain natriuretic peptide (BNP) mRNA expression. Purity of all samples was confirmed by measuring their 260/280 nm absorbance ratio using a Take3 multivolume plate in a Synergy HT microplate reader (BioTek Instruments). cDNA was reverse-transcribed from 1 μg of total RNA using the SensiFAST cDNA Synthesis Kit (BIO-65053, Bioline) and used for qPCR using the SensiFAST SYBR Lo-ROX Kit (BIO-94020, Bioline) in a QuantStudio 3 RT PCR System (Thermo Fisher Scientific). Data were analyzed by the 2−ΔΔCt method to estimate each gene’s mRNA expression. The primers specific for each gene were: 5′ CTCCAGAACAATCCACGAT 3′/5′ CTTGAACTATGTGCCATCTTG′ (BNP) and 5′ CGTGATTAGTGATGATGAACC 3′/5′ GAGCAAGTCTTTCAGTCCT 3′ (HPRT).

### Cardiomyocyte isolation

Mouse cardiomyocytes were isolated using the simplified, Langendorff-free method^19^. Briefly, the thorax was open to expose the heart after complete anesthesia. After flushing with EDTA buffer into the right ventricle, the heart was sequentially digested with EDTA buffer (in mM: 130 KCl, 5 NaCl, 0.5 Na_2_PO_4_, 10 HEPES, 5 glucose, 10 DBM, 10 taurine, 5 EDTA), perfusion buffer (in mM: 130 KCl, 5 NaCl, 0.5 Na_2_PO_4_, 10 HEPES, 5 glucose, 10 DBM, 10 taurine, 1 MgCl_2_), and collagenase buffer (in mg/ml: 0.5 collagenase II, 0.5 collagenase IV, 0.05 protease XIV; in perfusion buffer) into the left ventricle (LV). Constituent chambers (atria, LV, and right ventricle) were then separated and gently pulled into 1-mm pieces using forceps. Cellular dissociation was completed by gentle trituration, and collagenase activity was inhibited by addition of a 5 mL stop buffer (5% of SBF in perfusion buffer). Next, the cell suspension was passed through a 100-μm filter, and extracellular Ca^2+^ concentration was raised to 1 mM over four sequential rounds of gravity settling, using three intermediate Ca^2+^ reintroduction buffers (0.25, 0.5, and 1 mM). Finally, cells were collected and resuspended in Tyrode`s solution (in mM: 128 NaCl, 0.4 NaH_2_PO_4_, 6 glucose, 5.4 KCl, 0.5 MgCl-6 H_2_O, 5 creatinine, 5 taurine, 25 HEPES, 1.2 CaCl_2_) for further studies.

### Cellular volume

Cellular volume was evaluated following a previous report^20^. Briefly, cells were incubated with calcein-AM (5 µM, Life Technologies, Carlsbad, CA, USA) in Tyrode’s solution for 30 min at room temperature. Afterward, cells were rinsed with fluorophore-free Tyrode’s solution, and a stack of 2D images was taken every 1 µm in the z-axis covering the whole cell depth. The measurements were acquired using a Leica TCS SP5 confocal microscope equipped with a D-apochromatic 40×, 1.2 NA, oil objective (Leica Microsystems GmbH, Wetzlar, Germany), using a 488 nm excitation wavelength and a 500–600 nm emission window. Only rod-shaped cells with visible striation without spontaneous contractions were assessed. Analysis was carried out using ImageJ software (NIH). First, the pixel intensity frequency histogram corresponding to the calcein-AM signal was obtained. The sum of the pixel was multiplied by the pixel size and z-step between focal planes and expressed as cell volume.

### Cell shortening and Ca^2+^ transient measurements

Cell shortening and intracellular Ca^2+^ dynamics were evaluated simultaneously; images were obtained with a laser scanning confocal microscope in the line scan mode. The scanning line was longer than the cell length. Consequently, myocyte length shortening is reflected as an increase in empty space beyond the two longitudinal cell edges, following a previous report^21,22^. Briefly, a rectangular region comprising both cellular edges was selected, and a threshold was set to distinguish the intra-cellular from the extracellular space, converting the image into binary (Supplementary Fig. S1).

The cell border of the resulting binary image was compared to the border on the original record to ensure a close fit. *Cell shortening* is defined as the maximal difference between cell lengths. *Maximal shortening velocity* is defined as the first derivative of cell length tracing. *Time to peak shortening* (TTPS) is the time elapsed between the onset and the maximal shortening points, and *time to half relaxation* (TTHR) is the time elapsed between the maximal shortening time and the moment by which the cell length has recovered to 50% of the maximal shortening.

After isolating, cardiomyocytes were incubated in 10 µM Fluo-4 AM (F14201, Life Technologies, USA) in Tyrode’s solution (1 mM Ca^2+^) for 45 min at 25 °C. Afterward, cells were washed with fluorophore-free Tyrode`s solution and mounted in a stimulation chamber. The solution temperature in the stimulation chamber was maintained at 23 ± 2 °C. Confocal microscopy recorded line scan images taking a longer region than the cell longitudinal axis (for cell shortening, a pinhole optimized for a 4-µm-thick section in the focal plane was used, while a 1-µm-thick section was used for Ca^2+^ transient). Fluo-4 was excited at 488 nm, and the emission window was 500–600 nm. For cell shortening and Ca^2+^ transient, the cells were field stimulated by four electric pulses at each frequency 0.5, 1, and 3 Hz to achieve a quasi-steady state in Ca^2+^ transients as previously described^23^. The train of four pulses of 20 V/cm amplitude and 5 ms duration was generated using a function generator programmed in LabView that controlled an amplifier circuit through a data acquisition board (DAQ) (National Instruments) where the output signal of the circuit is connected to the stimulation electrodes localized in the field stimulation chamber RC-21BRFS (Warner Instruments). In each experiment, a specific value of frequency and noise is examined, the chamber is stimulated whit a pulse train, and the parameters corresponding to the contractile response and Ca^2+^ transients obtained from the third and four stimuli are averaged. The data are presented as the average of 40 different myocytes studied per animal. To analyze the noisy stimuli effect, three different Gaussian white noise levels with amplitude values (10, 20 and 30%) developed in LabVIEW, where the noise was added to the signal from the first to the fourth pulse (Supplementary Fig. S2). Baseline values correspond to the shortening and Ca^2+^ transient response without noise applied. Data analyses were performed in MATLAB version 9.11.0.1809720 (R2021b) (The MathWorks, Inc., Natick, MA). Intracellular Ca^2+^ dynamics were evaluated by characterizing transient amplitude and raise rate. *Ca*^*2*+^ *transient amplitude* is defined as the maximal F/F0 value, where F0 is the average fluorescence intensity before Ca^2+^ transient rise; *raise rate* is defined as F/F0/time to peak, where time to peak is the time between the onset and the peak value; *time to 50% of decay* (T_50%_) is the time elapsed between the peak and the moment the peak amplitude has decayed by 50%. All the confocal measurements were acquired using a Leica TCS SP5 confocal microscope equipped with a D-apochromatic 63×, 1.2 NA, oil objective (Leica Microsystems GmbH, Wetzlar, Germany).

### Mitochondrial membrane potential (ΔΨm) in cardiomyocytes

Freshly isolated cardiomyocyte cells were incubated with 300 nM TMRE (tetramethylrhodamine ethyl ester perchlorate) (T669, Thermo Fisher Scientific, USA) for 30 min at 25 °C in Tyrode’s solution supplemented with 1 mM Ca^2+^. Afterward, the cells were washed with a fluorophore-free Tyrode’s solution and mounted in a stimulation chamber. The solution temperature in the stimulation chamber was maintained at 23 ± 2 °C. 2D images (1024 × 1024 pixels, 400 Hz, 1 μm section thickness) were taken using 543 nm excitation and 555–700 nm emission window.

### Mitochondrial respiratory activity

Mitochondrial respiratory activity in permeabilized cells was determined by High-resolution respirometry (Oroboros instruments, Innsbruck, Austria). Two thousand cardiomyocytes were suspended in 2 mL of respiratory buffer (110 mM Sucrose, 60 mM potassium gluconate, 10 mM KH_2_PO_4_, 3 mM MgCl_2_, 0.5 mM EGTA, 20 mM HEPES pH 7.4, 0.1% BSA). After signal stabilization, cells were permeabilized with 10 µM digitonin. Succinate was employed as a respiratory substrate. State 3 (phosphorylating state), state 4, and uncoupled state were induced by adding 10 mM ADP, 2.5 µM oligomycin, and 1 µM FCCP, respectively. Non-mitochondrial respiration was obtained by the addition of 1 µM antimycin A. Respiratory control ratios (RCR) were obtained by dividing the mean value of respiration after ADP addition by the mean value after oligomycin addition. At the end of the experiment, cytochrome *c* oxidase (Cox) activity was determined by the sequential addition of 1 mM Ascorbate and 200 µM *N*, *N*′, *N*′-tetramethyl-p-phenylendiamine (TMPD) into the oxygraphy chamber. Cox activity was determined by subtracting the rate of oxygen consumption in the presence of TMPD/Ascorbate from the oxygen consumption induced by the autoxidation of TMPD. This last parameter was obtained by measuring the TMPD/Ascorbate-induced respiration inhibited by adding 50 µM potassium cyanide. Respiratory data were normalized to citrate synthase activity^24^. A previous titration protocol obtained optimal concentrations of detergent and respiratory modulators.

### Statistics

All data are presented as mean ± SEM. Student’s t-tests or Mann–Whitney U-tests were used to perform simple statistical comparisons. Two-way ANOVA followed by Tukey’s post-hoc was performed to analyze experimental groups with multiple comparisons. Differences were considered significant when *p* < 0.05. Data processing, graphs, and statistical analysis were performed with GraphPad Prism (GraphPad Software V.5.01; La Jolla, CA, USA).

## Results

As previously mentioned in the cardiovascular systems, the role of noise in cardiac cells still needs to be better understood. We studied how a noisy stimulus in cardiomyocytes from a cardiomyopathy model improves cardiac contractility and Ca^2+^ cell transients.

We performed experiments at different scales to validate the cardiomyopathy model. First, mice were administered DOX and checked for depression in cardiac function. Second, the atrophy of myocytes from DOX-treated mice was verified. Third, it was verified that the cellular response was diminished. The experiments are described in the following three sections.

### DOX treatment depresses cardiac function.

Cardiac function was assessed by pulsed doppler system to investigate the DOX effects at systemic level. Table 1 shows the cardiac function of both control and DOX-treated groups. Body weight, heart weight, heart weight-to-tibia length ratio, and heart rate decreased in the DOX-treated group. Aortic outflow is also referred to as LV ejection or systolic flow is compromised by DOX treatment. Likewise, we observed that peak aortic velocity, mean aortic velocity, ejection time, and peak acceleration were lower in the DOX-treated group.

**Table 1:** Animal description and Doppler measurements after DOX treatment.

Parameters	Unit	Ctrl (n = 9)		Dox (n = 11)	
Physical data					
Body weight	g	27.02 ± 0.5 (n = 5)		23.06 ± 0.5* (n = 6)	
Heart weight	mg	156.9 ± 8.6 (n = 5)		137.4 ± 5.4** (n = 6)	
Heart weight/Tibia length	mg/mm	8.3 ± 0.3 (n = 5)		6.3 ± 0.3 ** (n = 5)	
Aortic outflow data					
Heart rate	bpm	460 ± 8.4		362 ± 9.5***	
Peak aortic velocity	cm/s	117.2 ± 4.4		89.9 ± 2.8***	
Mean velocity	cm/s	22.31 ± 0.9		19.24 ± 0.4*	
Ejection time	ms	47.2 ± 1.7		53.1 ± 1.2*	
Peak acceleration	m/s^2^	188.9 ± 20.3		97.1 ± 5.4***	
Mitral inflow data					
Peak E velocity	cm/s	60.1 ± 1.8		51.9 ± 1.3*	
Peak A velocity	cm/s	40.4 ± 1.5		30.3 ± 3.2*	
E-A peak velocities ratio		1.4 ± 0.1		1.6 ± 0.2	

Parameters are means ± SEM.

*p < 0.05, **p < 0.001 and ***p < 0.0001 vs CTRL. Student t-test.

Furthermore, mitral inflow represents the flow from the left atrium into the LV during diastole. The mitral flow velocity signal is biphasic; one phase represents an early (E) peak during the rapid filling phase, while the other (A) peak occurs during atrial contraction. Thus, E and A peak velocities decreased in the DOX-treated compared to the control group. A representative Doppler displays of aortic and mitral signals are shown in the Supplementary Fig. S2.

These data suggest that mice treated with DOX led to a more pronounced reduction in aortic outflow velocity and a slight reduction in mitral inflow velocity which indicates low cardiac output or poor LV systolic function and low diastolic function, respectively; these effects depend on impaired cardiomyocyte function.

### DOX-induced cardiac atrophy

**Figure 1:**
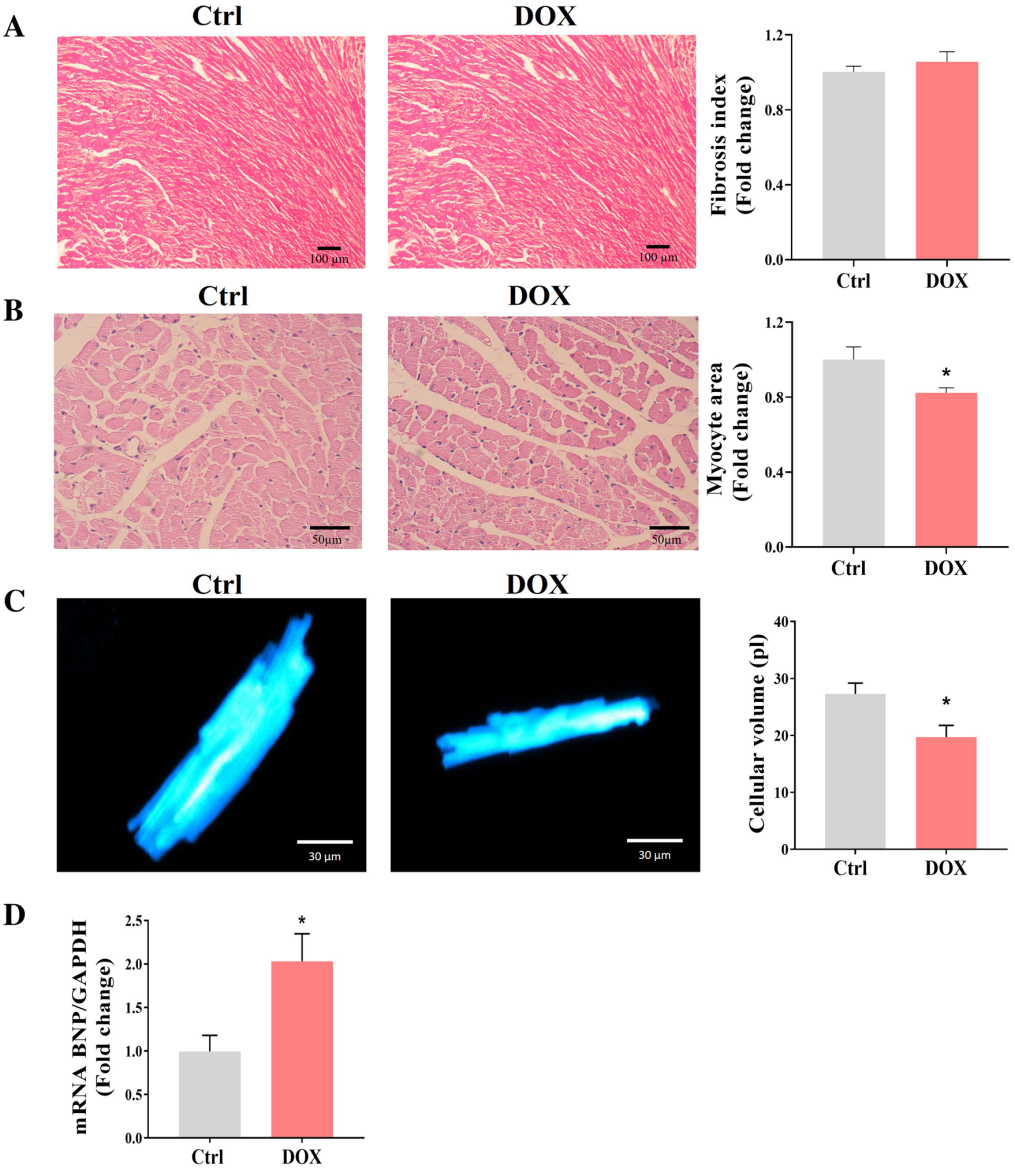
DOX treatment induces myocyte atrophy. (**A**) Representative ventricle stained with Masson’s trichrome (5×, left panel) and fibrotic index quantification (right panel). (**B**) Representative cross-section images of cardiomyocytes from control and DOX-treated groups (H&E, 10×, left panel) and myocyte area quantification (right panel). (**C**) Representative ventricle cardiomyocytes stained with calcein and analyzed by confocal microscopy (left panel) and cell volume analysis (Right panel). (**D**) qPCR analysis of BNP from the ventricular tissue samples. All data have been normalized to Ctrl mean values. The values are the mean ± SEM (n = 4 Ctrl and n = 6 DOX). Mann–Whitney U-tests; *p < 0.05 vs. Ctrl.

After the impaired cardiac function found in DOX-treated group, the characterization of the cardiac structure was performed. Histochemical analysis of collected hearts demonstrated no significant fibrosis in DOX-treated animals (Fig. 1A) as determined by Masson's trichrome staining. The cardiomyocyte area was analyzed using H&E-stained slides at the level of the papillary muscles, showing a 20% decrease in the DOX-treated group versus the control group (Fig. 1B). Concomitantly, we observed a significant reduction (30%) in the cellular volume of isolated ventricular cardiomyocytes in DOX-treated group compared to the control group (Fig. 1C). Gene expression of BNP, a relevant marker of cardiac hemodynamics, showed a two-fold increase in gene expression compared to the control group (Fig. 1D). These changes suggest that the DOX treatment induces cardiomyocyte atrophy which, partially contribute to the reduction in the ratio of heart weight to tibia length.

### DOX treatment compromises cellular shortening and Ca^2+^ transient

**Figure 2:**
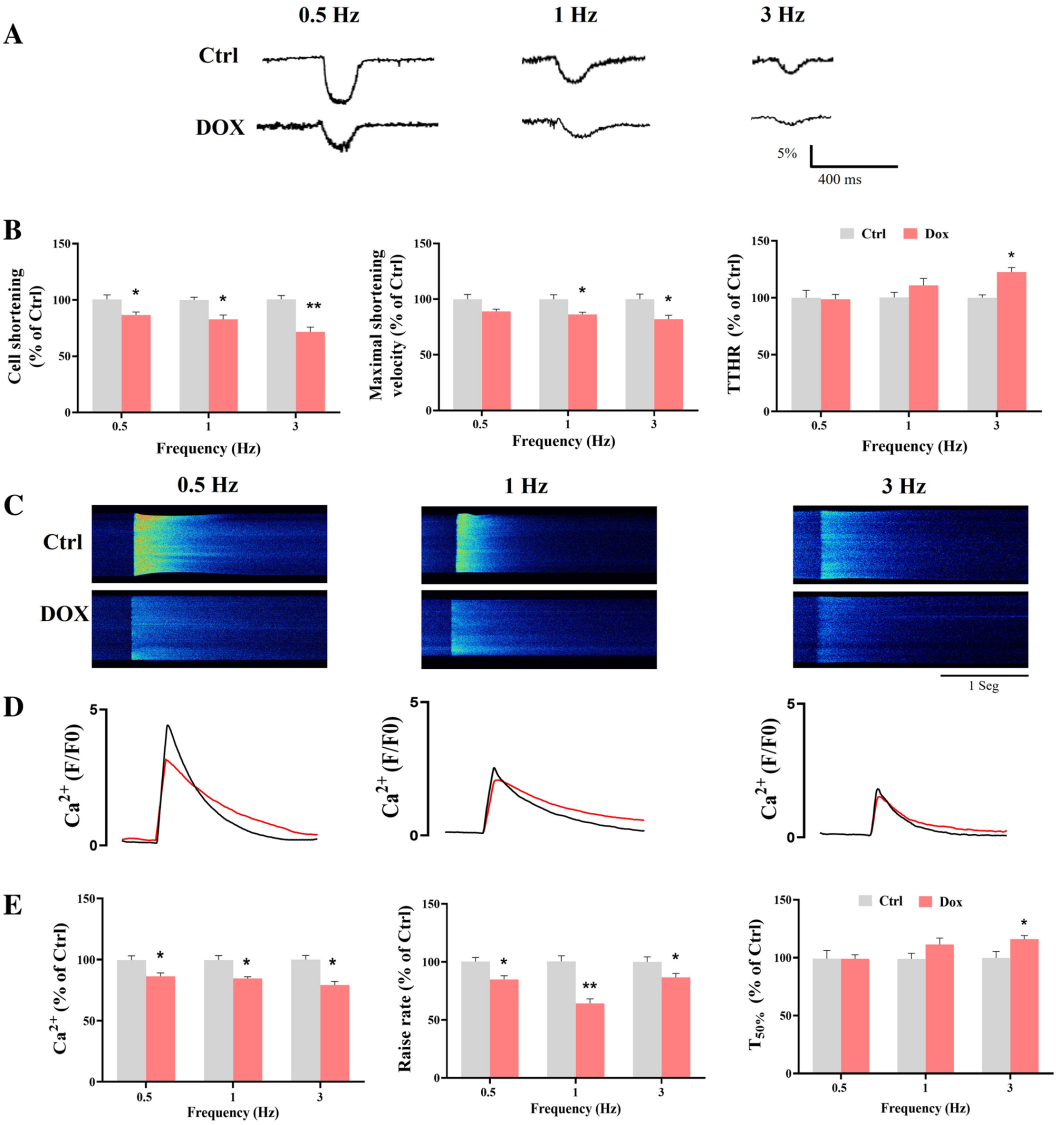
Cellular shortening and Ca^2+^ transient after DOX treatment. (**A**) Representative profile of cellular shortening at 0.5, 1, and 3 Hz. (**B**) Average cellular shortening, maximal shortening velocity, and TTHR at 0.5, 1, and 3 Hz. (**C**) Representative images of Ca^2+^ transient at 0.5, 1, and 3 Hz. (**D**) Representative fluorescence profile of Ca^2+^ transient. (**E**) Ca^2+^ transient amplitude, raise rate, and T_50%_ at 0.5, 1, and 3 Hz. Ctrl (black line) and DOX (red line). All data have been normalized to Ctrl mean values for each frequency. The values are the mean ± SEM (n = 6 Ctrl and n = 8 DOX). Students t-test; *p < 0.05 vs. Ctrl.

To characterize the effects of DOX-induced atrophy in the ECC, cell shortening and intracellular Ca^2+^ dynamics were evaluated under three stimulation frequencies (0.5, 1, and 3 Hz) in isolated cardiomyocytes. Cell shortening was characterized with the following parameters: cell shortening, maximal shortening velocity, and TTHR. The DOX-treated group showed compromised cellular shortening to all frequencies tested compared to the control group (Fig. 2A, B). A closer inspection showed that DOX treatment disrupted both phases of the contraction-relaxation cycle by decreasing the maximal velocity of shortening in 1 and 3 Hz paces; furthermore, it showed a statistically significant increase in time to half relaxation only in the 3 Hz pacing. TTPS was unaffected by DOX treatment (Supplementary Fig. S3). Cell contraction parameters in absolute units are reported in Table S1.

The calcium dynamics was characterized by the Ca^2+^ transient amplitude, time to peak, raise rate, and T_50%_ (Fig. 2C–E). Transient amplitude and raise rate decreased significantly at all paces in the DOX-treated group compared to the control group. No changes in time to peak were observed (Supplementary Fig. S3). However, T_50%_ only decreased at 3 Hz pacing in the DOX group compared to the control group. Our results shown that the decline in cardiac contraction after DOX treatment is due to a decline in both phases of the contraction-relaxation cycle, contributing to a less efficient cell contraction; while T_50%_ showed a tendency to increase, reaching significance only at 3 Hz. Our results show a disruption in the coordination of Ca^2+^ release, probably due to structural changes, might influence in the decreased Ca^2+^ transient amplitude, which compromises the cell contractility. Calcium dynamics parameters in absolute units are reported in Table S1.

### DOX induces cellular energetics failure

**Figure 3:**
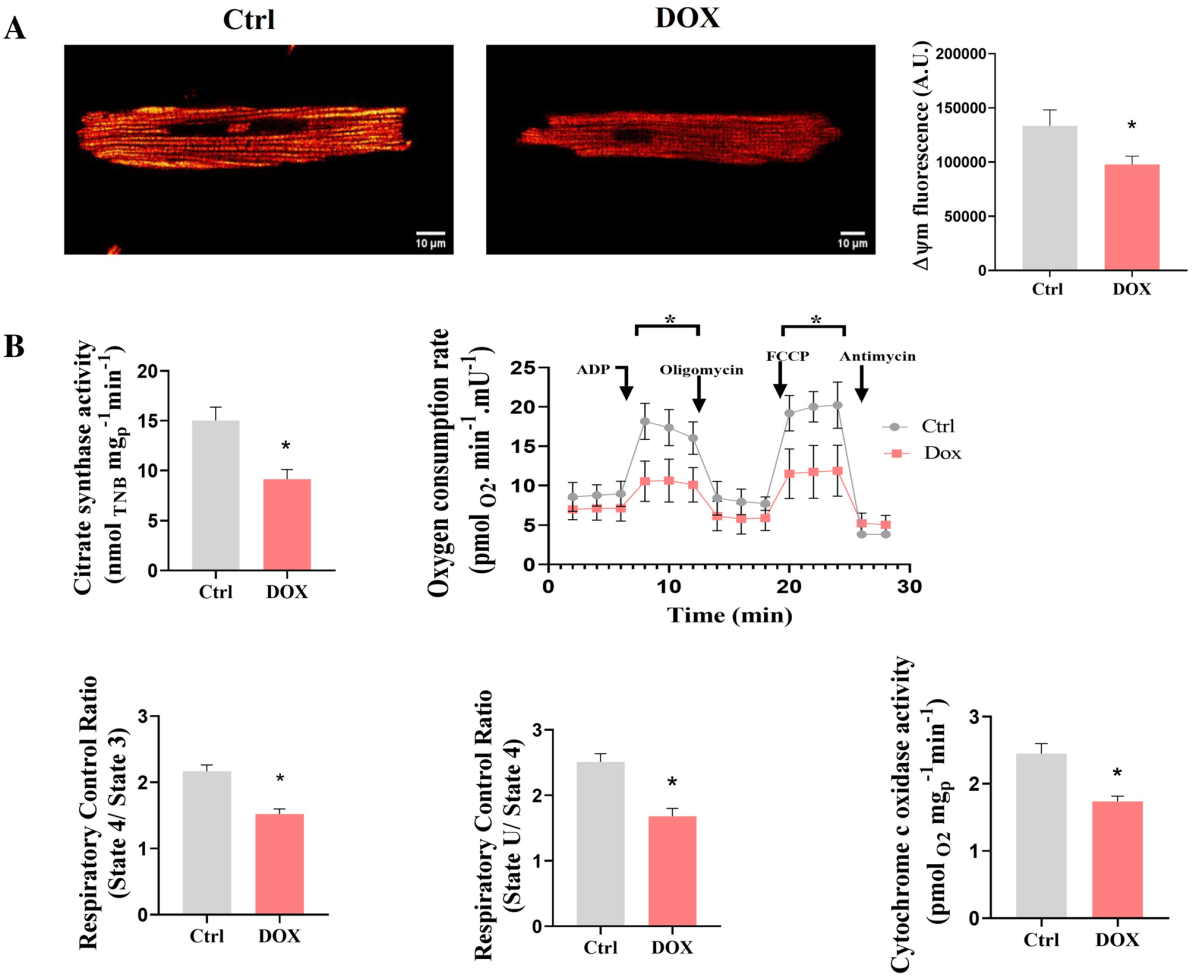
Mitochondrial function after DOX treatment. (**A**) Representative images from membrane potential (ΔΨm) in isolated cardiomyocytes. (**B**) Citrate synthase activity, oxygen consumption rate (OCR), respiratory control ratio (state4/state3 and state U/state 4), and cytochrome c oxidase activity (n = 5 Ctrl, n = 7 DOX). The values are given as the mean ± SEM. Mann–Whitney U-tests; *p < 0.05 vs. Ctrl.

Ca^2+^ handling is intimately linked to mitochondrial bioenergetics. Also, mitochondrial Ca^2+^ disturbance has been observed in DOX-treatment. For this reason, mitochondrial function was evaluated in isolated cardiomyocytes (Fig. 3). First, we measured the activity of citrate synthase, an established marker of mitochondrial mass in whole cells. The decreased activity of this marker enzyme indicates a lower mitochondrial mass in DOX-treated cells. Furthermore, we measured the respiratory activity in permeabilized cells to see if it correlates with the lower citrate synthase activity observed. As observed in Fig. 3B, a 2-fold decrease in phosphorylating and uncoupled respiration indicates that DOX treatment modifies the respiratory chain activity (Fig. 3B). Our results show that DOX-treatment unaltered basal oxygen consumption rates. In addition, we observed a diminished mitochondrial membrane potential (ΔΨm) indicating an energetic failure (Fig. 3A).

Moreover, these lower respiratory rates influenced the mitochondrial coupling, as observed in the respiratory control ratios in DOX-treated cells. These results indicate that some respiratory components cannot sustain high respiratory activities, but at rest, they present unaltered activities. This was corroborated when the activity of the terminal respiratory complex, cytochrome *c* oxidase (Cox) was measured. Thus, we observed that DOX-treated cells presented the lower activity of this enzyme, which indicates that this enzyme limits the respiratory activity only at high respiratory rates. Since all respiratory data were normalized to citrate synthase activity, we can rule out that the mitochondrial mass content per se affected the bioenergetic profile observed in our samples.

### Different intensities of noisy stimulus improve performance cellular shortening and Ca^2+^ transient after DOX treatment

We aimed to investigate whether a noisy stimulus affects the response of cardiomyocytes with impaired Ca^2+^ handling and contractile response. We perform experiments that electrically stimulate two cell groups: cardiomyocytes of DOX-treated mice as a cardiomyopathy model and cardiomyocytes from the control group. We registered cell shortening and Ca^2+^ dynamics when cell groups were electro-stimulated. Stimuli were applied at three frequencies with three noise levels added. Each data in DOX-treated animals was normalized by the data in control myocytes without noise to have a relative response to control myocytes.

**Figure 4:**
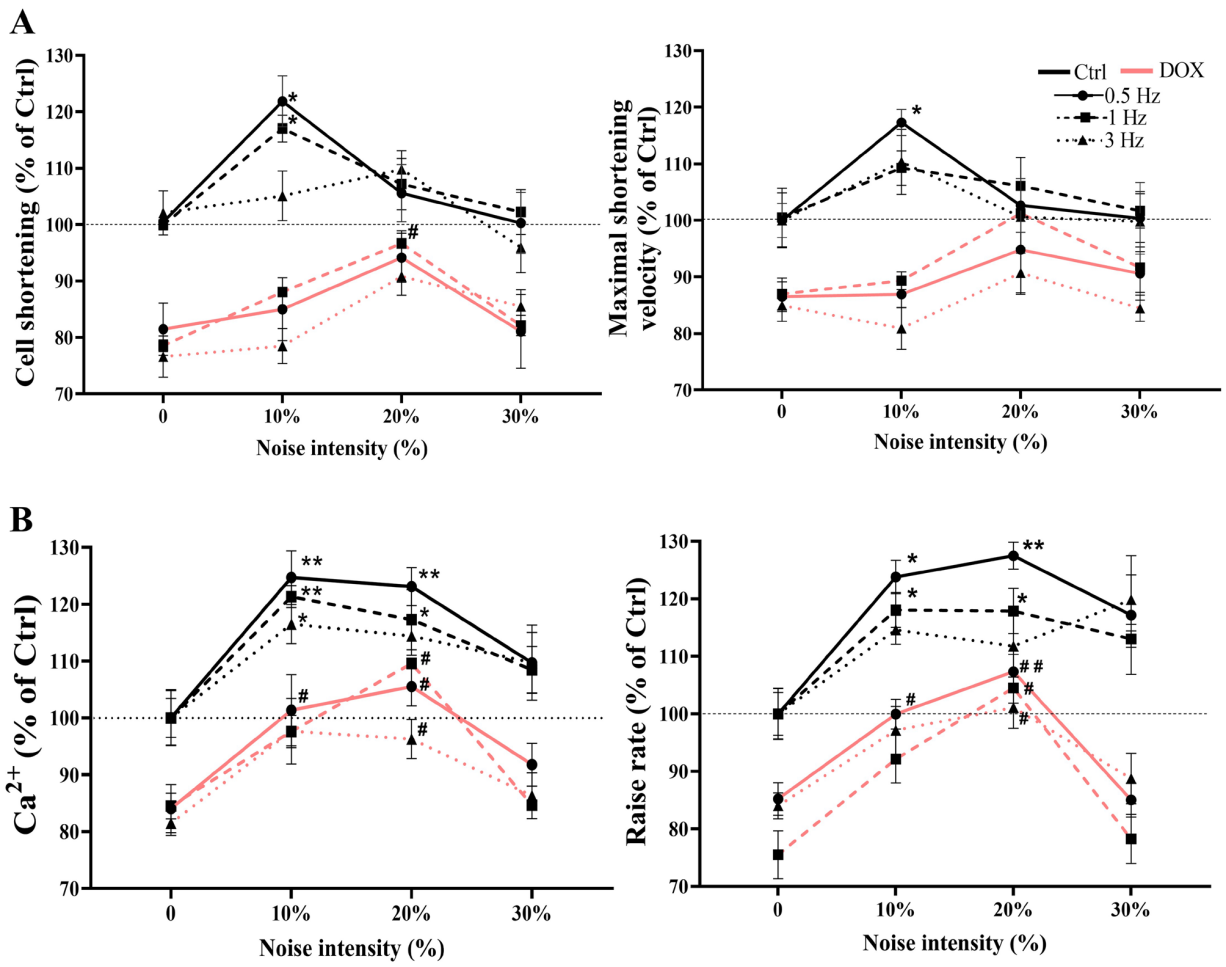
Effect of different intensities of noisy stimulus on the cellular shortening and Ca^2+^ transient after DOX treatment. (**A**) Cellular shortening and maximal shortening velocity at 0.5, 1, and 3 Hz for the different noisy levels. (**B**) Ca^2+^ transient amplitude and raise rate at 0.5, 1, and 3 Hz for different noisy levels. Ctrl (black line) and DOX (red line). Each data in the DOX-treated group was normalized by the data in control myocytes without noise. The values are the mean ± SEM (n = 6 Ctrl and n = 8 DOX). One-way ANOVA; *p < 0.05, **p < 0.01 vs. Ctrl 0% of noise; ^#^p < 0.05, ^##^p < 0.01 vs. DOX 0% of noise. Horizontal dotted lines represent the baseline level without noise in Ctrl myocytes.

Cell contraction was evaluated by characterizing cell shortening and maximal shortening velocity. We observed that cell shortening increases by 20% at 0.5 Hz and 16% at 1 Hz, with 10% noise-induced, while maximal shortening velocity increases by 17% at 0.5 Hz in control cells. In the absence of noise, cell shortening was 17% reduced, and maximal shortening velocity was 12% reduced in DOX-treated cells. Additionally, we found that 20% of noise-induced reach the control cell basal levels without noise in the stimulus. Maximal shortening velocity did not change compared with the control group (Fig. 4A). TTSP and TTHR were unaffected by noise addition on stimulus in both groups (Supplementary Fig. S4).

Since intracellular Ca^2+^ dynamics coordinate cell contractility, we evaluated Ca^2+^ dynamics by characterizing transient amplitude and raise rate. As noise level increases (10–20%), both Ca^2+^ transients and raise rate increase at 0.5 Hz in control myocytes. At 1 Hz, Ca^2+^ transient amplitudes increase as the noise level increases (10–20%). Raise rate increase with 10% noise level added. At 3 Hz, we observe that Ca^2+^ transients increase with 10% noise induced. Without noise in the stimuli, Ca^2+^ transient amplitude was 15% reduced, and the raise rate was 15% reduced in DOX-treated myocytes. As noise level increases in the range (10–20%), both Ca^2+^ transients and raise rate increases at 0.5 Hz. At 1 Hz, Ca^2+^ transient amplitudes and raise rate increase with a 20% noise level added. Additionally, we found that in the range of 10–20%, noise-induced cell response reaches the control cell basal levels without noise in the stimulus (Fig. 4B). Time to peak and T_50%_ were not affected by noise addition of stimulus in both groups (Supplementary Fig. S4).

## Discussion

Noise has recently emerged as a key component of a wide range of biological systems, from gene expression to heart function. Addition of white noise to electrical stimulation of isolated mouse heart enhances the electrical stimulation-contractile coupling response^14^. At cellular scale, noisy stimulation of H9c2 cells with a variable electric field produces modifications in the intracellular Ca^2+^ transient^16^. However, the noise role in cardiomyocytes with impaired contractile response and Ca^2+^ handling needs to be better understood.

Cardiac excitation–contraction coupling is the process from electrical excitation of the myocyte to contraction of the heart and it arises by the Ca^2+^-induced Ca^2+^-release mechanism^1^. Cardiomyocyte mishandling of Ca^2+^ is a central cause of both contractile dysfunction in pathophysiological conditions. The DOX-induced cardiac dysfunction model is a well-studied model over the years finding the mishandling of Ca^2+^ as one of the main pathophysiological features of this model^25,26^.

To study our hypothesis, we used a DOX-induced cardiomyopathy model. A dose of 25 mg/kg was applied following treatments reported by other authors^27,28^. It has been shown that cumulative dose regimens result in irreversible cardiomyopathy, steadily progressing toward congestive heart failure^29^. Our results show a significant change in heart rate and aortic blood velocities in DOX-treated mice. Likewise, cumulative treatment dose of DOX shows a dose-dependent decrease in LV ejection fraction^30^, which represents a hallmark of doxorubicin-induced cardiac dysfunction. According to pathophysiological changes, there was not tissue remodeling by fibrosis in ventricular wall, as neither the heart nor the myocytes were hypertrophied. However, we observed myocyte atrophy by histologic analysis and myocyte cellular volume semiquantification in the DOX group (Fig. 1). These effects were like those reported by Llach et al.^3^. Additionally, DOX treatment also increases the expression levels of BNP gene, a marker associated with impaired LV diastolic function during DOX therapy^31,32^.

We further analyzed myocyte response using confocal microscopy by assessing cell shortening and intracellular Ca^2+^ signaling. Contraction-relaxation cycle and it signaling pathway are well understood^1^. Our results shown that altered cellular contraction-relaxation induced by DOX treatment has a negative impact on the overall dynamic. There were alterations in SR Ca^2+^ release synchronicity during Ca^2+^ transient becoming evident by an increased raise rate, resulting in a delayed shortening at different frequencies stimulus (Fig. 2). Ca^2+^ release synchronicity provides a measurement of the reliability of systolic function. Likewise, the prolongation of raise rate is related to contractile dysfunction^33^ and DOX-induced cardiomyopathy^34^. The prolongation Ca^2+^ release time is associated with asynchronous Ca^2+^ release from RyR2 since this receptor plays a crucial role in prolonging the time of Ca^2+^ release. Previous studies have shown that DOX treatment reduces the expression of RYR2 in cardiomyocytes^35^ as well as increases the open probability of the channel, thereby resulting in increased calcium leak from the SR^36^, enhanced Ca^2+^ sparks occurrence and may be implicated in the decreased SR Ca^2+^ load^3^. Also, DOX-induced RyR2 Ca^2+^ leakage was associated with an enhanced production of ROS and apoptosis; therefore, the impact on Ca^2+^ transient dysregulation and ROS production augmented each other, leading to a vicious cycle^37^. Relaxation dynamics showed significant change only at 3 Hz, where DOX slightly prolongs cell relaxation (Fig. 2). T_50%_ prolongation of Ca^2+^ transient and TTHR are indicative of impairment in diastolic Ca^2+^ uptake by the SR and it is linked to diastolic dysfunction in cardiomyocytes of Dox-treated rats^38^. It has related that abnormal Ca^2+^ liberation by DOX could be induced by alteration of SERCA2a expression^3^. Additionally, structural changes in the cardiomyocyte due to cellular atrophy cause a disruption in the t-tubule distribution, altering the structure and modifying the distance between L-type Ca^2+^ channels and the RyR2 receptors, contributing to the impaired contractility^39^. We performed experiments to analyze the mitochondrial activity to explore whether severe cell damage caused by DOX is linked to the energy status by mitochondria. Our results shown that DOX-treated group led to a decreased in the mitochondrial membrane potential, the electrochemical force needed for ATP production, because of a lower activity of the mitochondrial respiratory chain (Fig. 3). Likewise, several studies have consistently reported that DOX administration has been related to mitochondrial dysfunction in cardiomyocyte^40–42^. For this reason, we hypothesized that cell Ca^2+^ mishandling induced by DOX treatment could related in part to mitochondrial dysfunction. Alterations in the mitochondrial membrane potential are influenced by several factors affecting the mitochondrial physiology like mitochondrial biogenesis or the activity of the respiratory chain. Previous reports indicates that DOX induce oxidative damage to mitochondrial components like lipids and proteins^43^. Cardiolipin is a mitochondrial phospholipid that is an early target of oxidant species, and acts as a key regulator of respiratory enzymes like cytochrome *c* oxidase or the F_1_F_o_-ATP synthase^44^. Our results agree with these authors.

Our next task was to investigate the noisy stimulus effect on the myocyte response. We electrically stimulated isolated cardiomyocytes from control and DOX-treated groups, in presence of added Gaussian white noise in the electrical signal (10, 20 and 30%) at 0.5, 1 and 3 Hz. Adding 10 and 20% of noise in the signal at 0.5 and 1 Hz in control cells, facilitates the Ca^2+^ release from the SR during the excitation–contraction coupling, resulting in Ca^2+^ transients with increased amplitude and raise rate compared with stimulation without noise added. These findings about Ca^2+^ transient are in agreement with results reported by Ramirez-Hurtado and colleagues. Their experiments, in H9c2 cells, show that low noise intensities favor amplitude and raise rate in Ca^2+^ dynamics^16^. Surprisingly, the DOX-treated cells responded to the levels of the control group despite their alterations in the respiratory chain. Adding 10 and 20% of noise in the stimuli induced a larger Ca^2+^ transient amplitude and increase raise rate, while the cell shortening increased only 20% of noise in the stimuli is added. It should be noted that this improvement was not the same level as the control group, but it did reach the baseline level. Also, the pacing was crucial since most of the effects were observed at low frequencies (0.5 and 1 Hz) (Fig. 4).

Our results show that both cellular contractility and Ca^2+^ handling are improved in control and DOX-treated myocytes. It might be associated with alterations in the sarcolemma Ca^2+^ channels/transporters such as L-type voltage-dependent Ca^2+^ (ICa,L), Na+/Ca^2+^ exchanger (NCX) or other molecules related to the release of Ca^2+^ transient; since it has been shown that DOX treatment does not affect the activity or the expression of ICa,L, and NCX^3,35,45^. Recently, Sciancalepore and colleagues reported that “noisy” electrical stimulations were already proved to be more effective in inducing firing, Ca^2+^ transient changes and contractions in cultured mouse myotubes^46^. Moreover, Bosutti and colleagues demonstrated that noisy stimulus favors the excitability of skeletal muscle cells and suggested that intrinsic variability characterizing the “noisy” might facilitate a cumulative membrane depolarization responsible for the Ca^2+^ release from the SR during the excitation–contraction coupling, resulting in Ca^2+^ transients with increased peaks and area^15^. Our results agree with these authors (Fig. 4). The stochastic resonance caused by noisy stimuli is related with the recruitment of ion channels. Onorato and colleagues observed increases on the spike firing in sensory neurons, where added external noise favors the recruitment of transient voltage-gated channel^13^. Therefore in our experiments, we propose that the noisy stimuli may induce fluctuations in the membrane potential, increasing the probability of opening of ICa,L and therefore, improving the intracellular Ca^2+^ dynamics even in myocytes with compromised cellular contractility, Ca^2+^ handling, and cellular energetics. We drew this conclusion with reservations, a detailed experimental analysis of the contribution of each voltage sensitive molecule such as ICa,L, NCX, and RYR2 of excitable cells stimulated with noisy electrical signal requires further investigation.

We can conclude from these facts that the noisy stimulation of cardiomyocytes with impaired Ca^2+^ handling and contractile response improves cell shortening, Ca^2+^ transient amplitude, and raise rate. Dox-treated cells reached the basal level of myocyte control with a specific noise level and frequency.

### Supplementary Information

Supplementary Information.

## Data Availability

The experimental data generated and analyzed during the study are available from the corresponding author upon reasonable request.
