# Supplementary material for: Cellular shortening and calcium dynamics are improved by noisy stimulus in a model of cardiomyopathy
Source: Sci Rep. 2023 Sep 9;13:14898. doi: 10.1038/s41598-023-41611-6 (PMC10492796; doi:10.1038/s41598-023-41611-6)
Supplement: Supplementary file 1 — Supplementary Information. [file 41598_2023_41611_MOESM1_ESM.docx]

**Supplementary Information**

**Cellular shortening and calcium dynamics are improved by noisy stimulus in a model of** **cardiomyopathy**

Russell Morales-Rubio ^a^, Judith Bernal-Ramírez ^b c^, Nestor Rubio-Infante ^b c^, Luis A Luévano-Martínez ^b c^, Amelia Ríos ^a^, Bruno A. Escalante ^a^, Gerardo García-Rivas ^b c^, Jesús Rodríguez González ^a †^

**^a^** Centro de Investigación y de Estudios Avanzados del I.P.N - Unidad Monterrey. Vía del Conocimiento 201, Parque de Investigación e Innovación Tecnológica. C.P.: 66600, Apodaca, Nuevo León, México.

**^b^** Tecnologico de Monterrey, Escuela de Medicina y Ciencias de la Salud, Cátedra de Cardiología. Hospital Zambrano Hellion, TecSalud, San Pedro Garza García, México.

**^c^** The Institute for Obesity Research, Tecnologico de Monterrey, Monterrey, Mexico.

^†^ **Corresponding author:**

Jesús Rodríguez-González,

Centro de Investigación y de Estudios Avanzados del I.P.N - Unidad Monterrey. Vía del Conocimiento 201, Parque de Investigación e Innovación Tecnológica. C.P.: 66600, Apodaca, Nuevo León, México.

E-mail: jrodriguez@cinvestav.mx Tel: +52 8111561740; Fax: +52 8111561741.


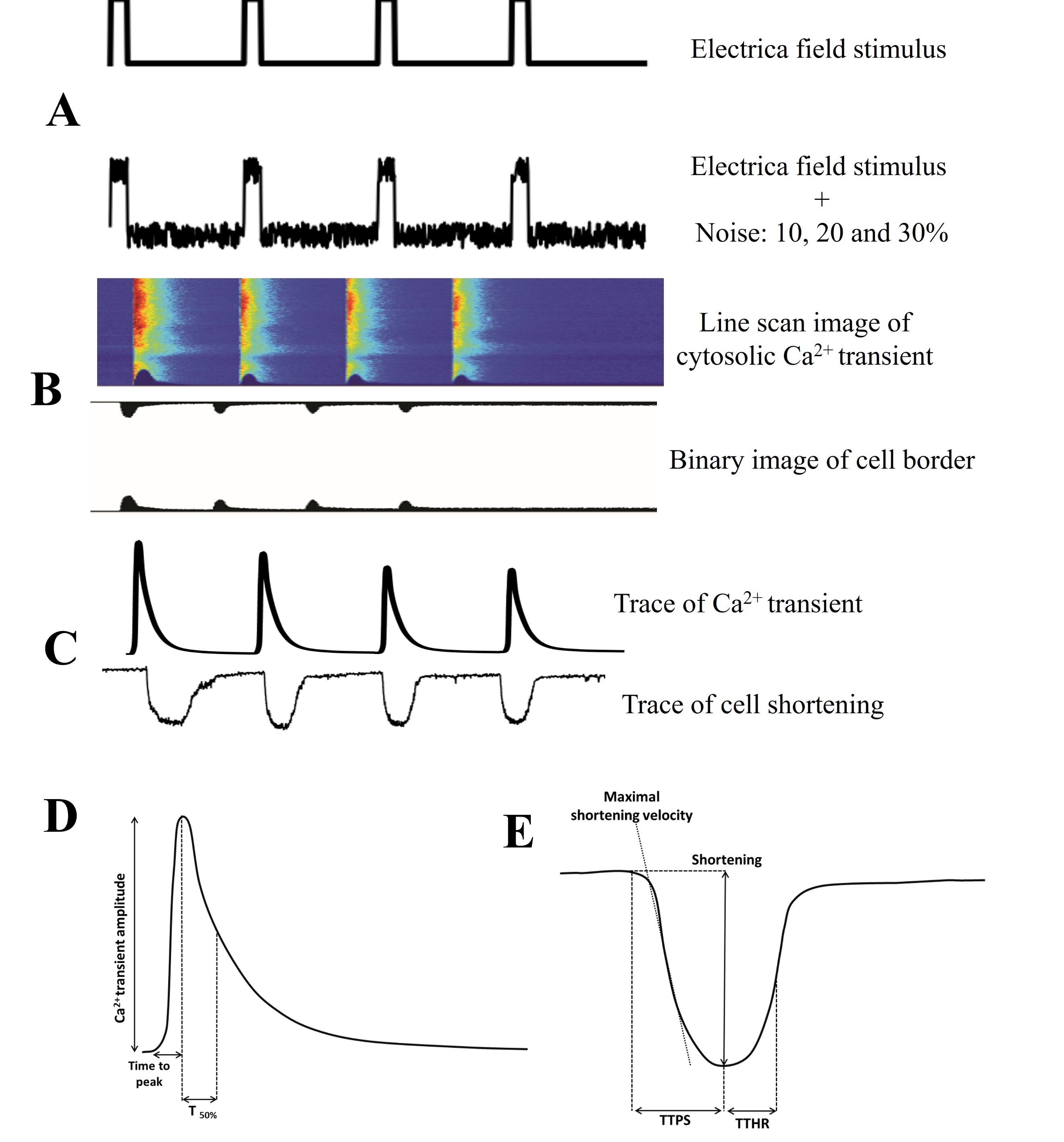


**Supplementary Figure S1**. Protocol of electric stimulation and Ca^2+^ transient and cell shortening measurements. A. Electric field stimulation by four pulses protocol at 0.5, 1, and 3 Hz and the protocol of electric field stimulation pulses with added Gaussian white noise with amplitude values (10, 20, and 30%). B. Representative confocal line scan image of cytosolic Fluo-4 fluorescence during steady-state field stimulation pulses and a binary image of cell border. C. Representative image of the time course of the cytosolic Ca^2+^ and the time course of the cell shortening. D. Parameters used to describe the Ca^2+^ transient: Ca^2+^ transient amplitude, time to peak, and time to 50% of decay (T_50%_). E. Parameters used to describe the cell shortening: cell shortening, maximal shortening velocity, TTPS, and TTHR.

**
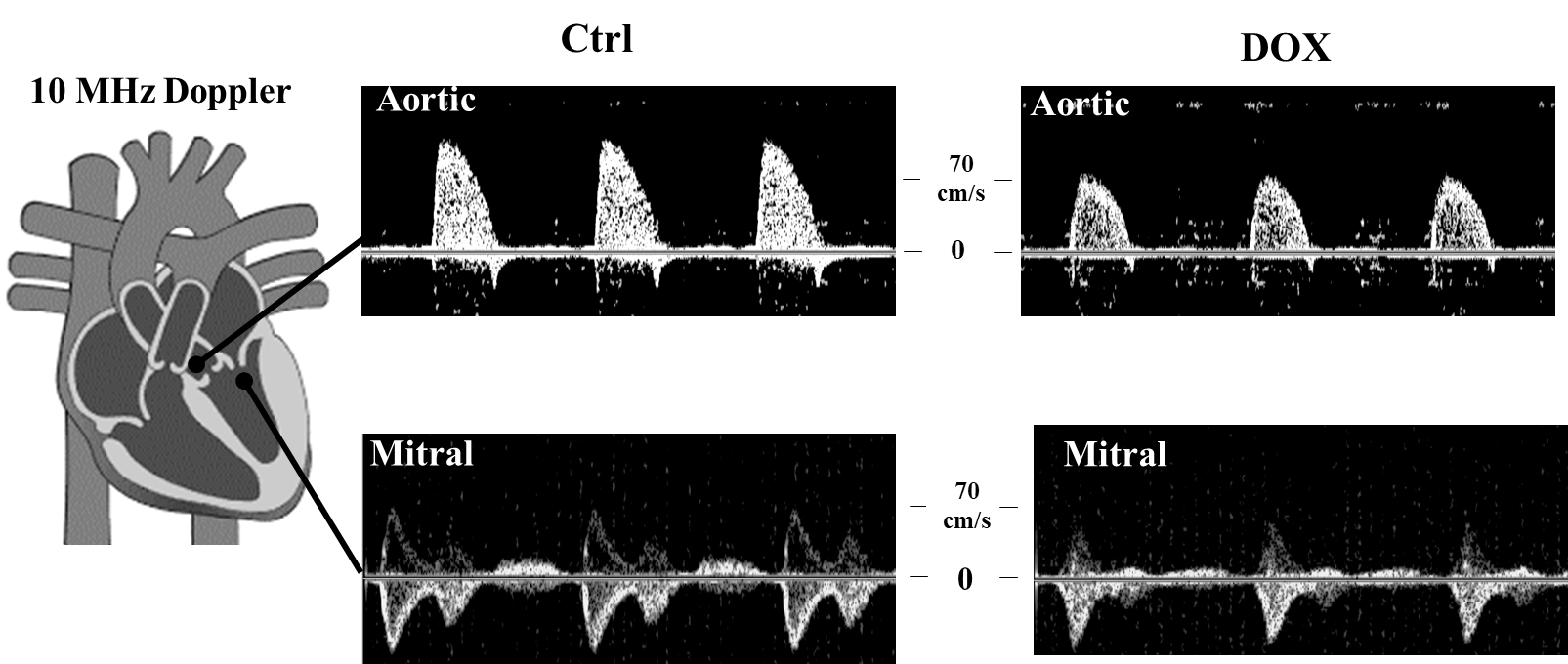
**

**Supplementary Figure S2.** Diagram and Doppler displays of aortic and mitral signals from Ctrl and DOX treatment mice using a 10-MHz probe positioned just below the sternum at the apex of the heart.


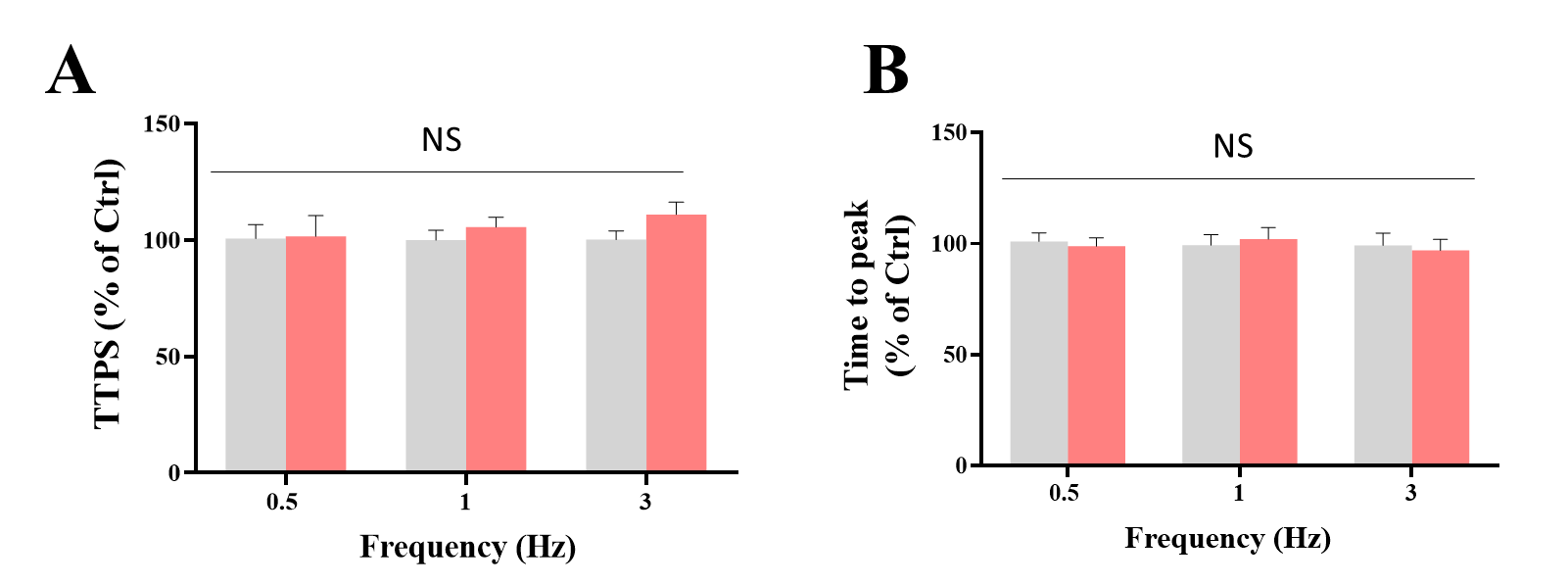


**Supplementary Figure S3.** Cellular shortening and Ca^2+^ transient after DOX treatment. A. Average TTPS at 0.5, 1, and 3 Hz. B. Average time to peak of Ca^2+^ transient at 0.5, 1, and 3 Hz. All data are normalized to Ctrl mean values for each frequency. The values are the mean ± SEM (n = 6 Ctrl and n = 8 DOX). Not significant (NS).

**Table S1**. Absolute units of cell shortening and Ca^2+^ transient parameters after DOX treatment

| **Parameters** | | **Unit** | **Ctrl (n=6)** | **DOX (n=8)** |
| --- | --- | --- | --- | --- |
|  |  |  |  |  |
| Cell Shortening | 0.5 Hz | % | 6.3 ± 0.91 | 5.2 ± 0.88 |
|  | 1 Hz |  | 5.2 ± 0.22 | 3.9 ± 1.33 |
|  | 3 Hz |  | 3.3 ± 0.23 | 2.5 ± 0.31 |
|  |  |  |  |  |
| Maximal shortening velocity | 0.5 Hz | µm/s | 85.86 ± 12.56 | 74.25 ± 13.56 |
|  | 1 Hz |  | 132.63 ± 4.01 | 122.67 ± 7.22 |
|  | 3 Hz |  | 92.28 ± 6.45 | 85.31 ± 7.17 |
|  |  |  |  |  |
| TTHR | 0.5 Hz | ms | 159.6 ± 31.19 | 146.37 ± 3.49 |
|  | 1 Hz |  | 124.5 ± 21.42 | 138.0 ± 13.11 |
|  | 3 Hz |  | 53.9 ± 15.42 | 64 ± 5.11 |
|  |  |  |  |  |
| Ca^2+^ amplitude | 0.5 Hz | a.u. | 3.4 ± 0.16 | 3.0 ± 0.20 |
|  | 1 Hz |  | 3.2 ± 0.22 | 2.8 ± 0.05 |
|  | 3 Hz |  | 3.3 ± 0.12 | 2.7 ± 0.06 |
|  |  |  |  |  |
| Raise rate | 0.5 Hz | a.u./s | 0.08 ± 0.12 | 0.064 ± 0.01 |
|  | 1 Hz |  | 0.12 ± 0.02 | 0.06 ± 0.01 |
|  | 3 Hz |  | 0.13 ± 0.03 | 0.1 ± 0.03 |
|  |  |  |  |  |
| T_50%_ | 0.5 Hz | ms | 350.1 ± 66.6 | 344.6 ± 22.22 |
|  | 1 Hz |  | 296.9 ± 22.6 | 351.1 ± 68.02 |
|  | 3 Hz |  | 185.0 ± 20.1 | 213.6 ± 24.12 |

Parameters are mean ± SEM


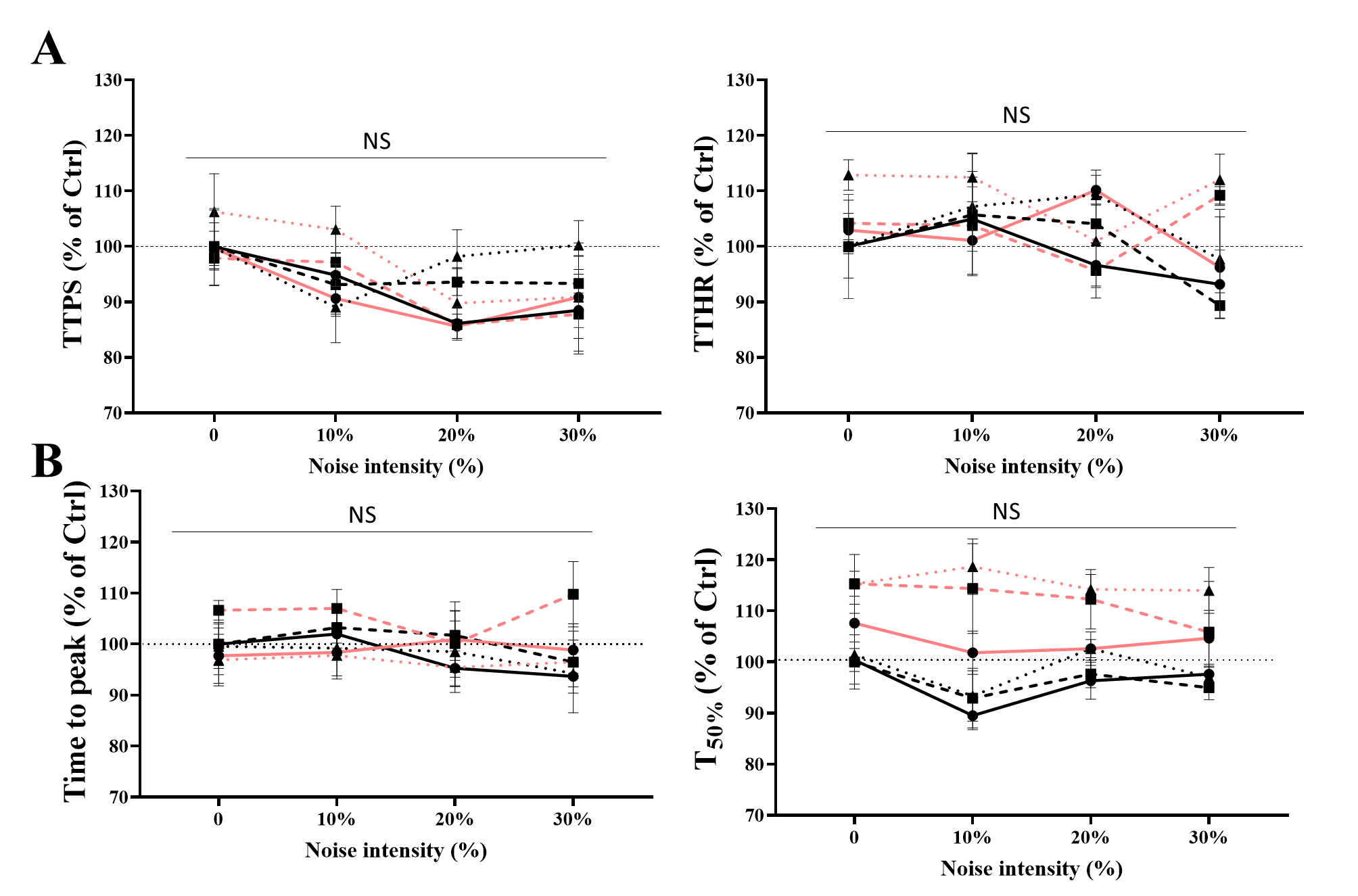


**Supplementary Figure S4.** Effect of different intensities of noisy stimulus on the cellular shortening and Ca^2+^transient after DOX treatment A. TTPS and THRR at 0.5, 1, and 3 Hz for the different noisy levels. B. Time to peak and T_50%_ of Ca^2+^ transient at 0.5, 1, and 3 Hz for different noisy levels. Ctrl (black line) and DOX (red line). Each data in DOX-treated animals was normalized by the data in control myocytes without noise. The values are the mean ± SEM (n = 6 Ctrl and n = 8 DOX). Not significant (NS). Horizontal dotted lines represent the baseline level without noise of Ctrl myocytes.
